# Inpatient Trauma Care Costs in the US From 2012 to 2021

**DOI:** 10.1001/jamanetworkopen.2025.33204

**Published:** 2025-09-23

**Authors:** Troy N. Coaston, Nam Yong Cho, Amulya Vadlakonda, Giselle Porter, Saad Mallick, Zeyu Liu, Sara Sakowitz, Peyman Benharash, Galinos Barmparas

**Affiliations:** 1 Center for Advanced Surgical and Interventional Technology, David Geffen School of Medicine at University of California, Los Angeles; 2 Department of Surgery, David Geffen School of Medicine at University of California, Los Angeles; 3 Department of Surgery, Cedars-Sinai Medical Center, Los Angeles, California

## Abstract

**Importance:**

Trauma care, with its inherent complexity and unpredictability, substantially contributes to health care costs in the US. Understanding temporal trends and associated factors may inform targeted cost-mitigation strategies.

**Objective:**

To examine trends in trauma-related inpatient costs from 2012 to 2021 and identify patient and hospital factors associated with contemporary costs.

**Design, Setting, and Participants:**

This retrospective cohort study used data from the 2012-2021 National Inpatient Sample, which captures 97% of US hospitalizations. Hospitalization for traumatic injuries were identified using *International Classification of Diseases, Ninth Revision* and *International Statistical Classification of Disease, Tenth Revision* codes for external causes of injury. The data analysis was performed between September 2 and October 28, 2024.

**Exposure:**

External causes of traumatic injury.

**Main Outcomes and Measures:**

The primary outcome was temporal trends in annual and per-patient hospitalization costs. Additionally, risk-adjusted associations of patient and hospital characteristics with inpatient costs in 2021 were assessed.

**Results:**

A total of 18 353 296 hospitalizations were identified during the study period (median [IQR] patient age ranging from 69 [47-83] years in 2012 to 70 [52-82] years in 2021; proportion of women ranging from 53.2% in 2012 to 50.7% in 2021). When stratifying by mechanism of injury, motor vehicle collisions incurred the highest median inpatient costs ($15 412; IQR, $8718-$29 376), followed by falls ($11 769; IQR, $6930-$19 052), other blunt trauma ($9818; IQR, $5567-$17 488), and penetrating injury ($9669; IQR, $4948-$19 545). In 2021, falls accounted for the largest share of costs (70.0%), while patients aged 75 years or older represented the most costly group (34.8%) and Medicare incurred the highest costs among all payers (52.6%). Annual inpatient trauma care costs increased from $27 billion in 2012 to $42 billion in 2021. Median per-patient costs rose from $10 662 (IQR, $6141-$17930) to $14 124 (IQR, $8249-$23 491). Following risk adjustment (2021), motor vehicle collisions (β = $4735.80; 95% CI, $4337.19-$5134.41 [reference, falls]), Black race (β = $1134.86; 95% CI, $628.07-$1641.67 [reference, White race]), and care in the Pacific region (β = $7763.20; 95% CI, $6176.90-$9350.31 [reference, New England]) were associated with greater hospitalization costs.

**Conclusions and Relevance:**

This cohort study found that inpatient trauma costs nearly doubled between 2012 and 2021, with geriatric falls a major contributor. Geographic and demographic disparities underscore the need for targeted interventions. Addressing systemic inefficiencies and standardizing care practices are critical to curbing rising costs while ensuring equitable trauma care.

## Key Points

**Question:**

What trends and factors were associated with inpatient trauma care costs in the US from 2012 to 2021?

**Findings:**

This cohort study of 18 353 296 hospitalizations found that national inpatient trauma costs nearly doubled over 10 years, with geriatric falls being the primary factor. Geographic and demographic disparities associated with higher costs were motor vehicle collisions, Black race, and care delivered in the Pacific region.

**Meaning:**

These findings suggest that with rising trauma-related costs, cost-saving strategies are needed to target inefficiencies and disparities within trauma systems to ensure sustainable and equitable care.

## Teaser

This cohort study examines the trends in US inpatient trauma care costs from 2012 to 2021 and identifies patient and hospital factors associated with contemporary costs.

## Introduction

Health care costs across the US increased by 4.1% in 2022 and now account for nearly 20% of the gross domestic product.^1^ Considering the unrelenting rise in health care costs, initiatives that include value-based reimbursement paradigms, improved care coordination, and longitudinal postdischarge follow-up have been devised to mitigate costs across specialties.^2,3,4^ Nonetheless, costs are projected to climb at a steady rate over the next decade.^5^

Trauma care, perhaps due to its inherent unpredictability, patient complexity, and costly interventions, is among the largest contributors to health care spending in the US.^6^ Notably, inpatient trauma care costs more than doubled between 2001 and 2011^7^ and now represents 25% of all inpatient hospitalization costs. Contemporary studies spanning geriatric, orthopedic, and critical care medicine similarly have reported a rise in costs.^8,9,10,11^ However, despite these systemic increases in costs, our understanding of overall trauma-related costs remains limited, as existing analyses have been largely siloed within individual specialties.^12,13^

In this study, we used a nationally representative cohort of patients with traumatic injuries to examine temporal trends in trauma-related hospitalization costs. Secondarily, we aimed to quantify the patient and hospital factors associated with contemporary inpatient trauma costs.

## Methods

This retrospective cohort study used data from the 2012-2021 National Inpatient Sample (NIS). Maintained as part of the Healthcare Cost and Utilization Project, the NIS is the largest national all-payer database in the US and provides accurate estimates for nearly 97% of hospitalizations using a survey-weighting methodology.^14^ Because of the use of deidentified data, this study was given an exemption from full review and the need for informed consent by the Institutional Review Board of the University of California, Los Angeles. The study adhered to the Reporting of Studies Conducted Using Observational Routinely Collected Data (RECORD) reporting guideline, which extends the Strengthening the Reporting of Observational Studies in Epidemiology (STROBE) reporting guideline.

All admissions entailing a diagnosis of external traumatic injury were identified using previously reported *International Classification of Diseases, Ninth Revision *(*ICD-9*) and* International Statistical Classification of Diseases, Tenth Revision* (*ICD-10*) codes.^15^ Only patients with external causes of traumatic injury were included in the analysis; other causes (burns, drowning, submersion) were excluded. Additionally, records missing key data, including age, sex, primary payer, and mechanism of injury, were removed from the analysis (5.7%).

Following survey weighting, patient characteristics (age, race and ethnicity [Asian, Black, Hispanic, White, other (other race, multiple races)], and insurance status), as well as hospital characteristics (teaching status and geographic region) are reported in accordance with the NIS data dictionary.^14^ Race and ethnicity were included as a variable because they are established social determinantsof health that influence disparities in health care access, outcomes, and expenditures. Mechanism of injury was identified using the appropriate *ICD-9* and *ICD-10* codes and grouped into penetrating injury, falls, motor vehicle collisions, and other blunt trauma. The van Walraven modification of the Elixhauser Comorbidity Index was used for evaluating change in comorbidity burden over time.^16^ The Comorbid Operative Risk Evaluation score was used to quantify the burden of comorbidities in all models (2021 admissions only) and provided enhanced risk stratification and calibration in surgical populations using machine learning.^17^ The severity of injuries was considered using the Injury Severity Score derived from *ICD-9* and *ICD-10* codes as reported by Clark et al.^18^ Receipt of a procedure was defined as undergoing any medical or surgical procedure as determined by *ICD-10* coding and was included in all models. Inpatient costs were calculated using hospital-specific cost-to-charge ratios and adjusted for inflation using the 2021 Personal Health Index.^19^

### Statistical Analysis

Data were analyzed between September 2 and October 28, 2024. Categorical variables are reported as group proportions, while continuous variables are reported as medians with IQRs or means with SDs, as appropriate. The adjusted Wald test was used for assessing the significance of intergroup differences. The significance of temporal trends was evaluated using the Cuzick nonparametric rank-based test (nptrend). To facilitate interpretation relevant to the contemporary era, multivariable linear regression was used to assess factors independently associated with hospitalization costs in 2021. Regression outputs are reported as β coefficients with 95% CIs. Statistical significance was set at α = .05. All statistical analyses were performed using Stata, version 18.0 (StataCorp LLC).

## Results

**Figure 1.**
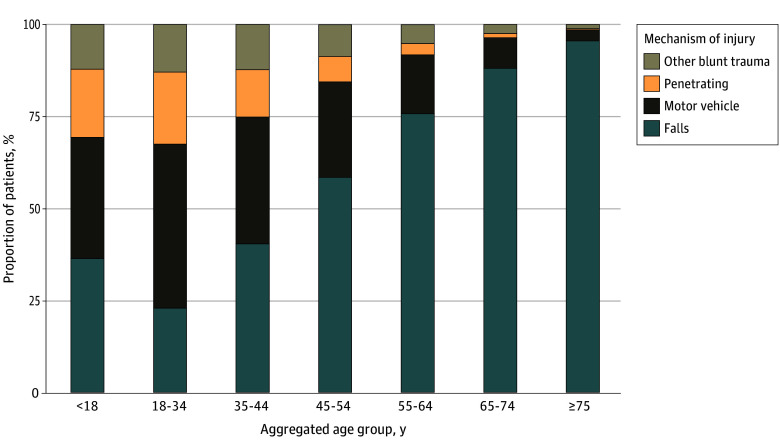
Primary Mechanism of Injury Stratified by Aggregated Age Group

A total of 18 353 296 hospitalizations for traumatic injury were identified from 2012 to 2021. Over the study period, significant increases in median age (from 69 years [IQR, 47-83 years] to 70 years [IQR, 52-82 years]), as well as the proportions of male patients (from 46.8% to 49.3%) and patients of Asian (from 1.7% to 2.1%), Black (from 8.5% to 10.8%), Hispanic (from 8.5% to 9.8%), and other (from 3.4% to 3.5%) race and ethnicity, whereas the proportions of female patients (from 53.2% to 50.7%) and White patients (from 72.6% to 71.3%) significantly decreased (all nptrend *P* < .001) (Table). Furthermore, the mean (SD) Injury Severity Score increased from 7 (6) in 2012 to 8 (9) in 2021 (nptrend *P* < .001), falls as the primary mechanism of injury rose in prevalence (from 73.3% in 2012 to 76.3% in 2021; nptrend *P* < .001), and a greater proportion of patients received care at teaching metropolitan centers (from 53.3% in 2012 to 77.3% in 2012; nptrend *P* < .001). When stratifying by age group, motor vehicle collision was the most common mechanism of injury among patients aged 18 to 34 years, while falls were the most common for all other groups (Figure 1).

**Table.** Trends in Demographic and Hospital Characteristics of Patients With Traumatic Injuries, 2012 to 2021

Characteristic	Patients, No. (%)			*P* value^a^
	Overall (N = 18 353 296)	2012 Only (n = 1 658 560)	2021 Only (n = 2 003 913)	
Age, median (IQR), y	70 (51-83)	69 (47-83)	70 (52-82)	<.001
Sex				
Female	9 547 043 (52.0)	882 580 (53.2)	1 015 729 (50.7)	<.001
Male	8 806 243 (48.0)	775 980 (46.8)	988 184 (49.3)	<.001
Elixhauser Comorbidity Index, median (IQR)^b^	3 (1-4)	2 (1-4)	3 (2-4)	<.001
Race and ethnicity				
Asian	365 200 (2.0)	28 045 (1.7)	42 760 (2.1)	<.001
Black	1 793 060 (9.8)	141 185 (8.5)	216 965 (10.8)	<.001
Hispanic	1 652 790 (9.0)	141 020 (8.5)	195 755 (9.8)	<.001
White	13 243 032 (72.2)	1 203 450 (72.6)	1 429 513 (71.3)	<.001
Other^c^	596 705 (3.3)	56 940 (3.4)	69 695 (3.5)	<.001
Missing	702 510 (3.8)	87 920 (5.3)	49 225 (2.5)	<.001
Income quartile				
76-100 (Highest)	3 794 919 (20.7)	359 810 (21.7)	421 320 (21.0)	<.001
51-75	4 340 139 (23.6)	384 780 (23.2)	478 715 (23.9)	<.001
26-50	4 690 814 (25.6)	404 910 (24.4)	494 475 (24.7)	.048
0-25 (Lowest)	5 137 924 (28.0)	469 950 (28.3)	568 979 (28.4)	<.001
Missing	389 500 (2.1)	39 110 (2.4)	40 425 (2.0)	<.001
Insurance coverage				
Private	3 616 329 (19.7)	367 195 (22.1)	366 545 (18.3)	<.001
Medicare	10 642 267 (58.0)	919 135 (55.4)	1 174 874 (58.6)	<.001
Medicaid	2 228 330 (12.1)	156 415 (9.4)	274 660 (13.7)	<.001
Other^d^	1 865 040 (10.2)	215 815 (13.0)	187 835 (9.4)	<.001
Injury Severity Score, mean (SD), units^e^	7 (8)	7 (6)	8 (9)	<.001
Mechanism of injury				
Fall	13 836 096 (75.4)	1 216 485 (73.3)	1 528 648 (76.3)	<.001
Motor vehicle collision	2 666 335 (14.5)	265 155 (16.0)	289 035 (14.4)	<.001
Penetrating injury	1 026 310 (5.6)	112 830 (6.8)	92 645 (4.6)	<.001
Other blunt trauma	822 615 (4.5)	64 090 (3.9)	93 585 (4.7)	<.001
Hospital bed size^f^				
Large	10 181 582 (55.5)	1 045 396 (63.0)	1 052 835 (52.5)	<.001
Medium	5 100 462 (27.8)	414 384 (25.0)	559 054 (27.9)	<.001
Small	3 069 491 (16.7)	198 780 (12.0)	392 024 (19.6)	<.001
Teaching status				
Nonmetropolitan	1 550 353 (8.4)	180 876 (10.9)	146 954 (7.3)	<.001
Nonteaching metropolitan	4 179 469 (22.8)	593 999 (35.8)	307 285 (15.3)	<.001
Teaching metropolitan	12 620 824 (68.8)	883 685 (53.3)	1 549 674 (77.3)	<.001

^a^ Nonparametric trend test.

^b^ Theoretical range is 19 to 89; however, clinically, scores fall between 0 and 40, with higher scores indicating a greater burden of comorbidities.

^c^ Includes other race or multiple races.

^d^ Includes self-pay, no charge, or other.

^e^ Scores range from 0 to 75, with higher scores indicating more severe injury.

^f^ Bed size categorization varies based on center location and teaching status (eg, large bed size in rural northeastern centers are at least 100 beds, whereas large bed size in midwestern rural hospitals are at least 50 beds).^20^

**Figure 2.**
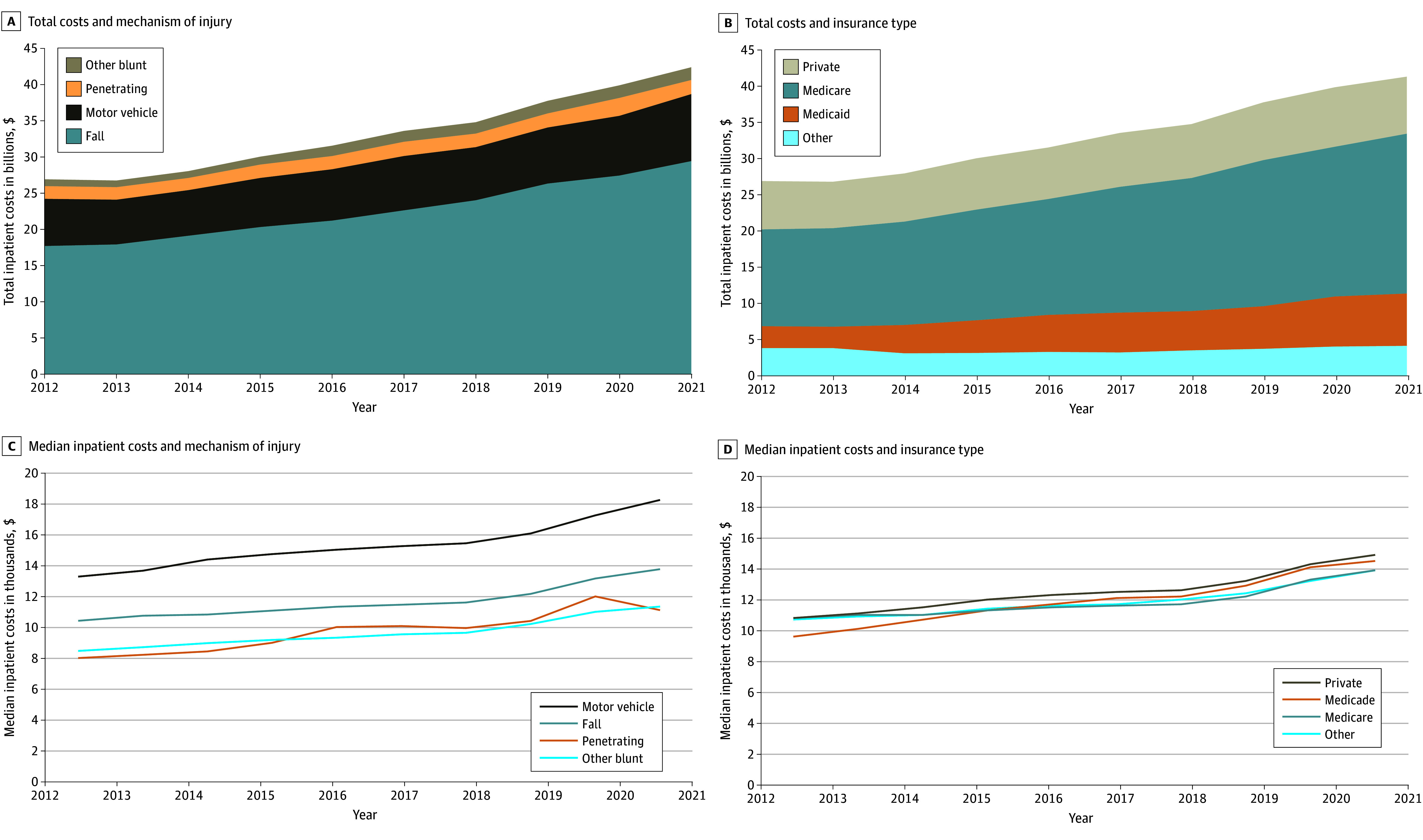
Trends of Inpatient Costs by Mechanism of Injury and Primary Payer

Over the decade analyzed, annual inpatient trauma care costs increased from $27 billion in 2012 to $42 billion in 2021 (nptrend *P* < .001). Moreover, median per-patient costs rose from $10 662 (IQR, $6141-$17 930) to $14 124 (IQR, $8249-$23 491) (nptrend *P* < .001). When stratifying by age, mechanism of injury, and primary payer, there was a notable rise in median costs across all categories, with the highest median inpatient costs for motor vehicle collisions ($15 412; IQR, $8718-$29 376; *P* = .004), followed by falls ($11 769; IQR, $6930-$19 052; *P* = .003), other blunt trauma ($9818; IQR, $5567-$17 488; *P* = .003), and penetrating injury ($9669; IQR, $4948-$19 545; *P* = .01) (Figure 2; eTables 1 and 2 in Supplement 1).

### Contemporary Inpatient Trauma Costs

**Figure 3.**
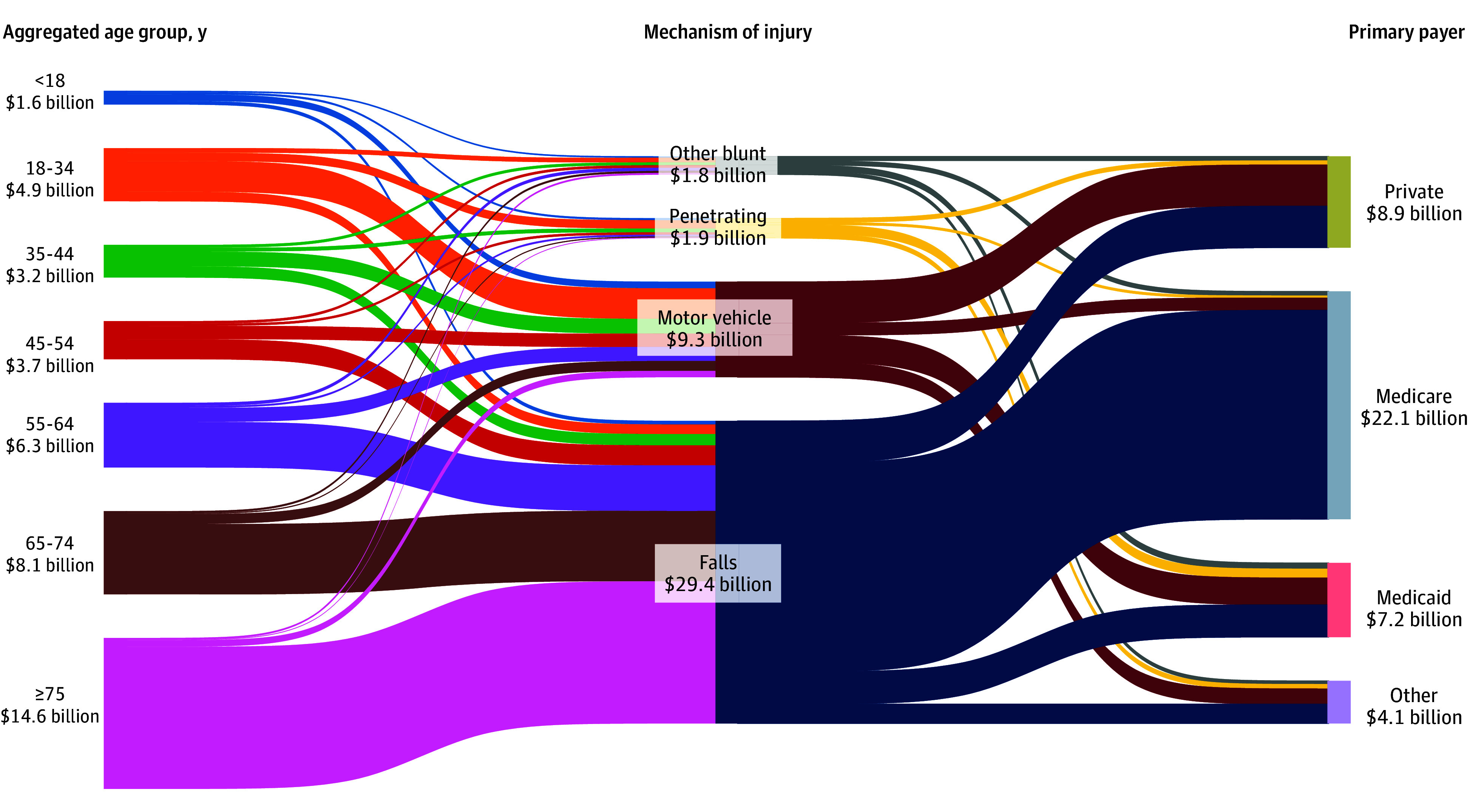
Hospitalization Costs Among 2021 Trauma Admissions

In 2021, patients aged 75 years or older (34.8%) accounted for the greatest estimated costs ($14.6 billion), while patients younger than 18 years (3.1%) had the lowest ($4.9 billion). When stratified by mechanism of injury, the majority of costs were from treating falls (70.0%) at $29.4 billion, while other blunt trauma (4.2%) had the lowest total costs at $1.8 billion. Finally, among all payers, Medicare beneficiaries accrued the highest costs (52.6%) at $22.1 billion, followed by privately insured patients (21.1%) at $8.9 billion and those with Medicaid (17.2%) at $7.2 billion (Figure 3).

**Figure 4.**
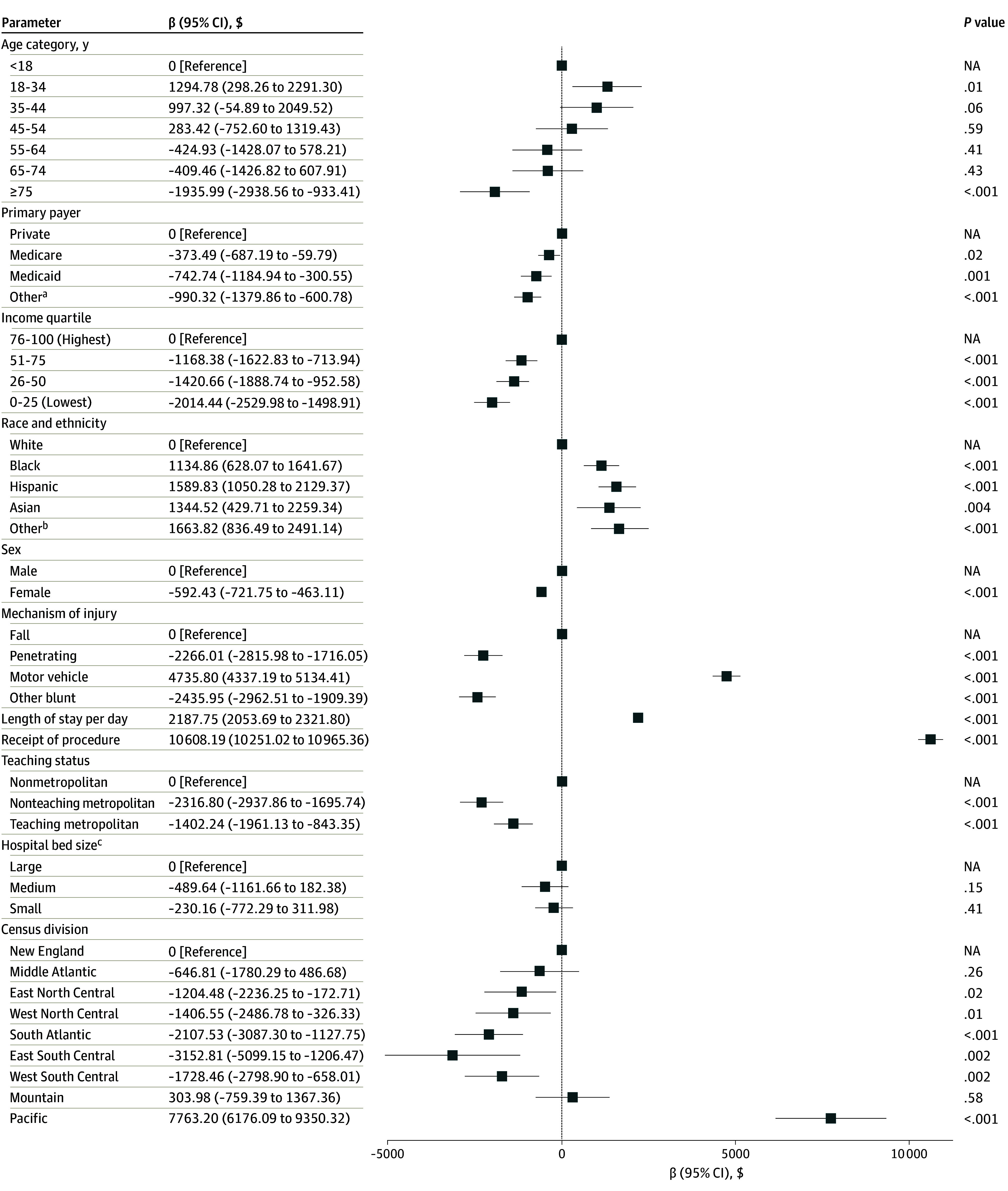
Risk-Adjusted Hospitalization Costs Among 2021 Trauma Admissions Covariates included all displayed parameters with the addition of injury severity (Injury Severity Score) and burden of comorbidities (Comorbid Operative Risk Evaluation score). NA indicates not applicable. ^a^Includes self-pay, no charge, or other. ^b^Includes other race or multiple races. ^c^Bed size categorization varied based on center location and teaching status (eg, large bed size in rural northeastern centers are at least 100 beds, whereas large bed size in midwestern rural hospitals are at least 50 beds).^20^

Following risk adjustment for factors including age, primary payer, income, race and ethnicity, sex, comorbidity burden, injury severity, mechanism of injury, length of stay, receipt of procedural intervention, center teaching status, hospital bed size, and US census division of center, motor vehicle collisions (β = $4735.80; 95% CI, $4337.19-$5134.41 [reference, falls]), Black race (β = $1134.86; 95% CI, $628.07-$1641.67 [reference, White race]), and care delivered in the Pacific US census division (β = $7763.20; 95% CI, $6176.90-$9350.32 [reference, New England]) were associated with increased inpatient costs. Conversely, female sex (β = −$592.43; 95% CI, −$721.75 to −$463.11), Medicare coverage (β = −$373.49; 95% CI, −$687.19 to −$59.79 [reference, private insurance]), and penetrating injury (β = −$2266.01; 95% CI, −$2815.98 to −$1716.05 [reference, falls]) were associated with a decrement in costs (Figure 4).

## Discussion

This retrospective cohort study analyzing 2012-2021 NIS data of inpatient trauma care costs found that both overall and per-patient costs increased, with a large proportion of spending attributable to geriatric falls. Several factors were associated with contemporary inpatient costs following risk adjustment, including geographic region, center teaching status, and race and ethnicity. These findings warrant further discussion.

We noted an increase in hospitalization costs among patients with traumatic injuries over the study period. Importantly, such an increase appeared to be independent of growing rates of traumatic injuries, considering that the per-capita costs only increased by 3% per year while overall spending grew by 4.5% annually. This finding is consistent with greater costs reported across a variety of surgical specialties.^21,22^ While overall contemporary cost trends among patients with traumatic injuries remain undescribed, a rise has been reported specifically among those with head, eye, and spinal injuries.^12,23,24,25^ A considerable share of hospitalization costs are medically necessary and probably unavoidable. A single-center study by Fakhry et al^26^ reported that while individuals with traumatic injuries only represented 7% of patients, those requiring intensive care unit stays longer than 10 days amassed nearly half of all trauma hospitalization costs. Cost-cutting strategies should instead target extraneous expenses. For example, among individuals sustaining traumatic brain injury, a recent systematic review of inpatient costs found inpatient length of stay to be a major driver of expenditure.^27^ Although multifaceted, the findings of Hwabejire et al^28^ suggested that prolonged length of stay may be driven by systemic issues, including rehabilitation facility placement, operational delays, and insurance coverage, rather than clinical factors. Addressing these systemic issues may be the ideal strategy for reducing inpatient costs.

A significant proportion of all inpatient trauma admissions and costs were associated with geriatric falls. It is estimated that by 2050, nearly 40% of patients with traumatic injuries will be older than 65 years.^29^ The reason for this high estimate is multifactorial. First, the US population is aging, with recent projections estimating that 1 in 5 individuals will be aged 65 years or older by 2030.^30^ Second, multimorbidity and polypharmacy predispose older patients to falls and subsequent deleterious sequelae.^31^ Moreover, older patients represent a particularly high-risk group for traumatic injury, as age has been previously associated with higher rates of intensive care unit use, nonhome discharge, and inpatient mortality.^32^ Plainly, the substantial cost burden of geriatric falls on the trauma system appears to be necessary; however, there are several promising strategies for cost reduction. Prior literature has suggested that older patients with traumatic injuries are undertriaged, which may be associated with unconscious bias, unreliable vital signs, and the often low-energy mechanism of injury seen among this population.^31,33^ Such misdiagnosis may ultimately incur greater costs when these patients require transfer for a higher level of care.^33^ In recent years, however, age-specific triage criteria have emerged with adequate efficacy.^31,34^ Another promising strategy is injury prevention. Indeed, a fall prevention program by Dykes et al^35^ boasted $4 million in savings across 2 health systems annually and was projected to save $1.8 billion if implemented nationally. Although these interventions are promising, geriatric falls could continue to be a substantial fiscal burden on trauma systems, and further study is required to attenuate costs.

Following risk adjustment, several factors, including geographic region, center teaching status, and race and ethnicity, were independently associated with incremental index hospitalization costs. It is well known that location, facility type, and differences in case mix introduce variation in inpatient costs across centers.^15,36,37^ For trauma care specifically, variation in trauma readiness costs, such as trauma team activation fees, may contribute to observed regional differences.^38,39^ In a 2024 national analysis, Scott et al^37^ found that centers in the western US had a $2000 higher trauma team activation fee than the next most expensive region. Since activation fees are determined by individual health systems, such charges may also contribute to cost variation at the center level. Indeed, Ashley et al^40^ found that the average annual readiness cost of level I trauma centers, which are commonly teaching hospitals, were more than double that of level II centers. This finding is consistent with ours of increased costs at teaching facilities. Additionally, our findings of increased costs among patient race and ethnicity other than White further align with previous reports.^23,41,42^ A retrospective study of more than 2500 patients with traumatic lower-extremity orthopedic injuries found that race and ethnicity other than White were associated with a twofold higher odds of catastrophic health expenditures.^42^ Importantly, the guaranteed coverage for catastrophic health expenditures within the Patient Protection and Affordable Care Act has been shown to reduce the racial and ethnic disparity among patients with traumatic injuries, suggesting that insurance-based disparities may be a driver of differences.^41^ The complex interplay among regional practices, facility characteristics, and patient factors has a substantial influence on trauma-related costs and should be further evaluated to develop targeted interventions.

### Limitations

This study had several limitations. First, as a large administrative database, the NIS is subject to undercoding and erroneous coding in addition to differential coding practices between centers. Additionally, year-to-year changes in the number of hospitals included in the NIS may influence annual estimates. Second, we used billing codes to estimate inpatient costs using the only available national inpatient cost data for trauma care to our knowledge. However, this approach may have overestimated costs, as both for-profit and not-for-profit hospitals possess financial incentives to optimize billing practices, thereby inflating reported costs.^43^ Third, we could not account for laboratory values in risk-adjusted models. Importantly, trauma center designation, costs, and charges are all influenced by state-level legislation differences, which similarly could not be included in the adjustment. Finally, our cost estimates differed from previous reports,^12^ which may have been influenced by the use of different cost indices. We used the Personal Health Care Expenditure component of the National Health Expenditure Accounts for the normalization of costs to 2021 US dollars, consistent with Healthcare Cost and Utilization Project recommendations.^19^

## Conclusions

In this cohort study, a nationally representative sample of patients with traumatic injuries was used to characterize temporal trends and contemporary patterns of inpatient costs. We found that hospitalization costs among trauma admissions were increasing, nearly doubling over the 10-year study period. Moreover, a large proportion of trauma-related costs was among elderly patients with fall-related injuries. Finally, several factors were independently associated with contemporary costs following risk adjustment, including geographic region, center teaching status, and race and ethnicity. With the cost burden of trauma increasing, additional effort to mitigate and identify potential cost-saving strategies is essential for ensuring a sustainable health care system.

## Notes

Supplement 1. **eTable 1.** Trends in Median Hospitalization Costs Among Patients With Traumatic Injuries Stratified by Aggregated Age Group **eTable 2.** Trends in Median and Cumulative Hospitalization Costs Among Patients With Penetrating Injury Stratified by Mechanism

Supplement 2.
Nonauthor Collaborators

Supplement 3.
Data Sharing Statement
