# Supplementary material for: Inpatient Trauma Care Costs in the US From 2012 to 2021
Source: JAMA Netw Open. 2025 Sep 23;8(9):e2533204. doi: 10.1001/jamanetworkopen.2025.33204 (PMC12457974; doi:10.1001/jamanetworkopen.2025.33204)
Supplement: Supplement 1. — eTable 1. Trends in Median Hospitalization Costs Among Patients With Traumatic Injuries Stratified by Aggregated Age Group eTable 2. Trends in Median and Cumulative Hospitalization Costs Among Patients With Penetrating Injury Stratified by Mechanism [file jamanetwopen-e2533204-s001.pdf]

Supplemental Online Content

Coaston TN, Cho NY, Vadlakonda A, et al. Inpatient trauma care costs in the US from 2012 to 2021. *JAMA Netw Open*. 2025;8(9):e2533204. doi:10.1001/jamanetworkopen.2025.33204

eTable 1. Trends in Median Hospitalization Costs Among Patients With Traumatic Injuries Stratified by Aggregated Age Group

eTable 2. Trends in Median and Cumulative Hospitalization Costs Among Patients With Penetrating Injury Stratified by Mechanism

This supplemental material has been provided by the authors to give readers additional information about their work.

© 2025 Coaston TN et al. *JAMA Network Open*.

eTable 1. Trends in Median Hospitalization Costs Among Patients With Traumatic Injuries Stratified by Aggregated Age Group

All values adjusted for inflation to 2021 US dollars.
Nptrend; non-parametric trend. IQR; interquartile range

Aggregated Age Group (years; median [IQR])	*Overall*	*2012 Only*	*2021 Only*	Nptrend
<18	$8000 [$4000-$15000]	$7000 [$4000-$13000]	$10000 [$5000-$20000]	<0.001
18-34	$12000 [$7000-$23000]	$11000 [$6000-$20000]	$15000 [$8000-$28000]	<0.001
35-44	$12000 [$7000-$23000]	$11000 [$6000-$19000]	$15000 [$8000-$27000]	<0.001
45-54	$12000 [$7000-$22000]	$11000 [$6000-$19000]	$15000 [$8000-$27000]	<0.001
55-64	$13000 [$7000-$22000]	$11000 [$7000-$19000]	$15000 [$9000-$26000]	<0.001
65-74	$13000 [$8000-$21000]	$11000 [$7000-$19000]	$15000 [$9000-$24000]	<0.001
≥75	$12000 [$7000-$19000]	$11000 [$6000-$17000]	$14000 [$8000-$21000]	<0.001

© 2025 Coaston TN et al. *JAMA Network Open*.

eTable 2. Trends in Median and Cumulative Hospitalization Costs Among Patients With Penetrating Injury Stratified by Mechanism

All values adjusted for inflation to 2021 US dollars.
Nptrend; non-parametric trend. IQR; interquartile range

	Overall (n=1,026,310)	***2012 Only*** (n=112,830)	***2021 Only*** (n=92,645)	**Nptrend**
Median Costs ([IQR])				
Stab Wounds	$8,000 [4000-14000]	$7,000 [4000-12000]	$9,000 [5000-17000]	<0.001
Gun Shot Wounds	$18,000 [10000-35000]	$15,000 [7000-30000]	$21,000 [11000-39000]	<0.001
Cumulative Costs				
Stab Wounds	$9,210,000,000	$963,000,000	$1,000,000,000	0.30
Gun Shot Wounds	$9,840,000,000	$805,000,000	$970,000,000	0.04

© 2025 Coaston TN et al. *JAMA Network Open*.
