# Supplementary material for: Inpatient Trauma Care Costs in the US From 2012 to 2021
Source: JAMA Netw Open. 2025 Sep 23;8(9):e2533204. doi: 10.1001/jamanetworkopen.2025.33204 (PMC12457974; doi:10.1001/jamanetworkopen.2025.33204)
Supplement: Supplement 2. — Nonauthor Collaborators [file jamanetwopen-e2533204-s002.pdf]

Supplemental Online Content: Nonauthor Collaborators
*First name, last name, and suffix (if applicable) are required and will appear in PubMed.

| *Group Name(s): Academic Trauma Research Consortium (ATRIUM) | | | | | | | |
|---|---|---|---|---|---|---|---|
| ***First Name and Middle Initial(s)** | ***Last Name** | ***Suffix (eg, Jr, III)** | Academic Degrees | Institution | Location (city, state/province, country) | Role or Contribution, eg, chair, principal investigator | Group (if more than 1 Group listed in the byline) and/or Subgroup (eg, Steering Committee) |
| Umar | Bhatti | | MD | Cedars Sinai | Los Angeles, CA, USA | Assistant | |
| Megan | Brenner | | MD | UCLA | Los Angeles, CA, USA | Advisor | |
| Oh Jin | Kwon | | MD | UCLA | Los Angeles, CA, USA | Assistant | |
| David | Machado-Aranda | | MD | UCLA | Los Angeles, CA, USA | Investigator | |
| Aricia | Shen | | MD | Cedars Sinai | Los Angeles, CA, USA | Assistant | |
| Areti | Tillou | | MD | UCLA | Los Angeles, CA, USA | Advisor | |
| Zachary | Tran | | MD | Vanderbilt | Nashville, TN, USA | Advisor | |
