# Supplementary material for: Inpatient Trauma Care Costs in the US From 2012 to 2021
Source: JAMA Netw Open. 2025 Sep 23;8(9):e2533204. doi: 10.1001/jamanetworkopen.2025.33204 (PMC12457974; doi:10.1001/jamanetworkopen.2025.33204)
Supplement: Supplement 3. — Data Sharing Statement [file jamanetwopen-e2533204-s003.pdf]

Data Sharing Statement

Data
Data available: No

Additional Information
Explanation for why data not available: These data are available for purchase from the Healthcare Utilization Project.
